# Complex hidradenitis suppurativa on a background of long‐standing Crohn's disease requiring radical pelvic and perineal reconstruction: A case report

**DOI:** 10.1002/ccr3.9203

**Published:** 2024-07-19

**Authors:** Bea Harris Forder, Sabina Nistor, Roman Mykula, Mark Bignell, Hooman Soleymani majd

**Affiliations:** ^1^ Medical Sciences Division University of Oxford Oxford UK; ^2^ Department of Gynaecology Oncology Oxford University Hospitals Foundation Trust Oxford UK; ^3^ Department of Plastic Surgery Oxford University Hospitals Foundation Trust Oxford UK; ^4^ Department of Colorectal Surgery Oxford University Hospitals Foundation Trust Oxford UK

**Keywords:** chronic diseases, gastroenterology/hepatology, obstetrics/gynecology, surgery

## Abstract

**Key Clinical Message:**

A surgical MDT approach to high‐complexity surgeries can allow maximal resection in order to achieve disease control and excellent functional outcomes, as demonstrated here for a case of hidradenitis suppurativa in a patient with Crohn's disease.

**Abstract:**

Hidradenitis suppurativa is an autoimmune disease characterized by abscess and fistula formation with purulent discharge in intertriginous zones, and is associated with inflammatory bowel disease. We present the case of a patient with severe ongoing hidradenitis suppurativa causing osteomyelitis and affecting the perineum, on a background of Crohn's disease previously treated with panprotocolectomy and permanent ileostomy. The hidradenitis suppurativa was having a severe impact on the patient's quality of life, and she had failed to respond to conservative management. The patient opted for a radical two‐step procedure: first her coccyx and sacrum were removed. The second step was a radical bilateral anterior vulvectomy and posterior vaginectomy, with preservation of the uterine body and cervix. An anterolateral thigh flap was used to reconstruct the perineum. This complex procedure required the expertise of multiple surgical specialties, including plastic, general, spinal, and gynecological oncology surgeons to achieve maximal disease resection, minimizing the risk of recurrence.

## 1 BACKGROUND

Crohn's disease is a form of inflammatory bowel disease (IBD) characterized by symptoms such as diarrhea, weight loss, abdominal pain, fatigue, anemia, and perianal fissures or fistulae.^1^ Inflammatory bowel disease has the highest incidence in Europe, having been suggested to affect up to 0.8% of the population, with its incidence rising worldwide.^2^ The management IBD is both medical, using immunosuppressants and disease‐modifying drugs, and surgical, with surgeries performed to remove parts of diseased bowel, with or without stoma formation. There are also many extraintestinal manifestations of IBD; these include arthropathies, hepatobiliary issues such as primary sclerosing cholangitis, eye conditions such as anterior uveitis and dermatological conditions such as erythema nodosum.^3^

More recently, it has been evidenced that there may be link between Crohn's disease and another dermatological condition, hidradenitis suppurativa,^4^, ^5^ an inflammatory skin disorder targeting apocrine glands, hence most often found in intertriginous areas where these glands are concentrated. Hidradenitis suppurativa is characterized by severe pain, purulent discharge and tissue destruction with scar development in affected regions; this can have profound psychosocial impact on individuals suffering with the disease.^6^, ^7^ Hidradenitis suppurativa is typically managed through a combination of lifestyle changes and antibiotic regimens, however in severe cases systemic immunomodulators or surgery may be required.^8^

We present a severe and complex case of hidradenitis suppurativa in a patient with a history of Crohn's disease. Her disease distribution differed from the typical skip lesions, rather it affected the entire colon (pancolitis). The disease was refractory to medical treatment, hence had been treated with a panprotocolectomy and end ileostomy The hidradenitis suppurativa was affecting her perineum, buttocks, natal cleft, bilateral thighs, perivagina, and labia, causing her immense pain, detriment to quality of life due to the extent of discharging sinuses, and severe threat to life due to repeated admissions with acute systemic sepsis requiring surgical debridement. The patient opted for maximal resection of these areas. The severity and large area of presentation required input from multiple surgical specialties through staged operations in order to remove the diseased areas while minimizing loss of healthy structures and allowing rehabilitation.

## 2 CASE PRESENTATION

We present the case of a woman in her 40s who has an extensive medical and surgical history following a diagnosis of Crohn's disease in her 20s. This was treated with a panprotocolectomy and permanent end ileostomy with a pedicled gracilis flap reconstruction. Since that procedure, the patient has not required treatment for her Crohn's disease. The patient then developed hidradenitis suppurativa, requiring a bilateral mastectomy to treat disease in the breast region. She subsequently developed disease in her perineum, buttocks, natal cleft, bilateral thighs, perivagina, and labia, causing ongoing purulent discharge from this area. Following this diagnosis, she underwent surgery for removal of perineal disease with a vertical rectus abdominis musculocutaneous flap reconstruction of the perineum. However, the hidradenitis suppurativa disease recurred within the area and extended into the previous flap donor sites.

Her medical history is significant for stage III chronic kidney disease, the cause for which is not certain, although the consensus is that this is likely to have occurred as an extraintestinal manifestation of Crohn's disease, as a result of the use of nephrotoxic drugs, or a combination of these factors. She has been menopausal for a couple of years, taking hormonal replacement therapy for the management of menopausal symptoms; her only other medications are sertraline and pregabalin. The patient is allergic to ciprofloxacin, metronidazole, adalimumab, azathioprine, and ibuprofen. The patient has a body mass of index of 24.81 kg/m^2^ and is an ex‐smoker having given up smoking in her 30s.

## 3 INVESTIGATIONS

Following repeated septic episodes the patient was referred from another unit to the plastic surgery department, who referred the patient for joint multidisciplinary review with dermatology, general surgery, and gynecology. Upon initial examination during consultation with a plastic surgeon, it was found that the patient had extensive 30 × 30 cm chronic scarring, induration, sinus formation in proximal bilateral thigh, groins, natal cleft, perineum, and bilateral labia majora. Within the area of previously resected anus and vertical rectus abdominis musculocutaneous flap and gracilis flap reconstructions, there was a granulating wound 16 × 6 cm, confluent with the areas of hidradenitis suppurativa. There were obvious discharging points at the superior aspect of the natal cleft, posterior vagina, perineum, left labia, and left medial thigh at the most proximal element of the healed gracilis scar left thigh.

The patient had undergone an extensive series of imaging for her hidradenitis suppurativa, with MRIs over multiple years showing the evolution of the disease, forming an increasing number of abscesses, pus collections, and fistulae. The most recent MRI prior to her surgery demonstrated changes consistent with osteomyelitis of the sacrum and coccyx, which was thought to be the origin of her septic episodes.

Blood tests identified an anemia, likely of chronic disease, which can be associated with hidradenitis suppurativa^9^ and Crohn's disease.^10^ To treat this, the patient received preoperative blood transfusions.

## 4 TREATMENT

There was extensive discussion between the patient and plastic surgeons regarding the merits of surgical management, in the form of coccygectomy and partial sacrectomy to excise the osteomyelitis, and radical bilateral anterior vulvectomy and posterior vaginectomy, with preservation of uterine body, and cervix. This was weighed up against conservative management in discussion with the patient. The patient's wishes were for maximal resection as she felt her quality of life had deteriorated significantly due to the constant wound discharge and pain, and wanted the diseased area removed. Due to the location of deep seated abscess in the presacral region, the extensive hidradenitis suppurativa involvement of the surrounding structures extending anteriorly and posteriorly, and consideration of postoperative wound care of flap reconstructions and rehabilitation, a staged approach was adopted. The first procedure would resect the osteomyelitis and deep abscess with sacrectomy and coccygectomy and partial closure of wound, with involvement of spinal surgeons, general surgeons, gynecological oncology surgeons, and plastic surgeons. The second procedure, a radical bilateral anterior vulvectomy and posterior vaginectomy with preservation of uterine body and cervix and flap reconstruction, required gynaecological oncology surgeons, general surgeons, and plastic surgeons.

**FIGURE 1:**
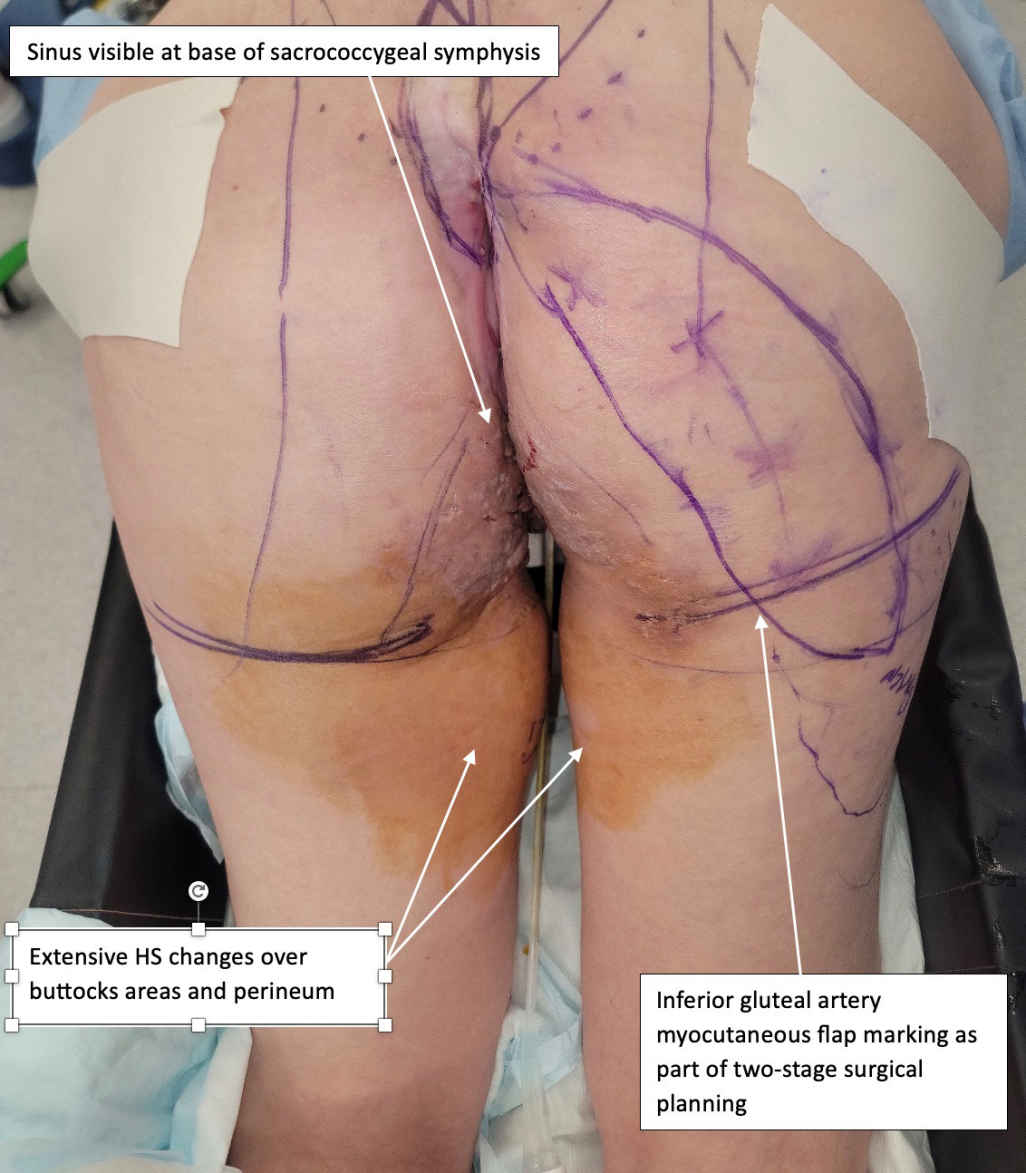
First procedure: Coccygectomy and partial sacrectomy: Patient in prone position, on Jackson table in a sling.

**FIGURE 2:**
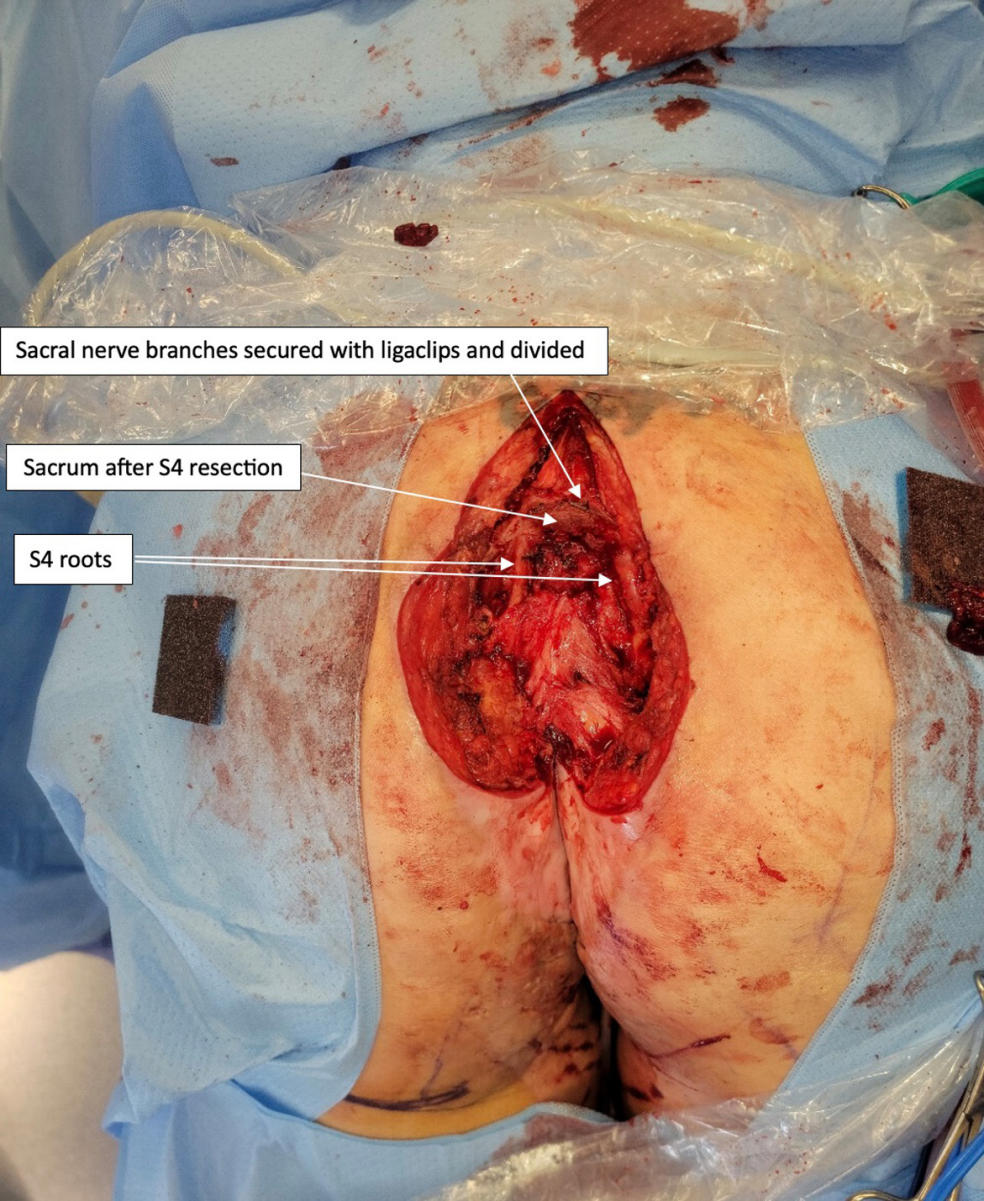
First procedure: Coccygectomy and partial sacrectomy.

For the first procedure, the patients was repositioned prone on a Jackson table in a sling in order to flex the hips and knees, exposing the perineum.^11^ Examination under anesthesia by the plastic, general and gynecological surgeons showed multiple sinuses and fistulae, some of which were healing and some which appeared chronic, consistent with longstanding hidradenitis suppurativa (Figure 1). There was no normal vulval tissue, although the clitoris, posterior vagina, urethra, paraurethral, ectocervix, and endocervical canal were normal. An elliptical excision was made surrounding the visible sinus at the base of the coccyx, this was extended up over the lower sacrum in the midline. The surgeons next dissected down to the posterior sacrum, leaving the excised skin and sinus tract attached to the coccyx. On dissection around the sacrum, there was pus identified on the right at the level of S5, which appeared to be coming from the posterior foramen. Dissection was extended to S4, with laminectomy performed at this level and the canal space entered. When encountered, nerve roots were secured with ligaclips and divided in order to preserve the exiting S4 nerve root. Further osteotomy cuts were made though S4 to join the presacral tissues, freeing the bony connections of S4 and the coccyx as a single specimen. The specimen was retracted to allow access to the anterior presacral soft tissues; this revealed the S4 nerve roots, which were encased in scar tissue but seen to run inferolaterally, and preserved. A pus collection was seen anterior to the sacrum. The surgeons continued to dissect through the indurated presacral scar tissue to release the soft tissue connections attached to the specimen, and the S4 was then enucleated through to the coccyx, along with the overlying skin (Figure 2). Further biopsies were taken of perineum and vulva to exclude malignancy of chronic wounds. It was decided not to proceed with closure of posterior natal cleft and sacral wound with inferior gluteal artery myocutaneous flap due to the extent of infection. The wounds were then partially advanced and sutured closed, and a negative pressure dressing applied at 100 mmHg, with plans for this to be changed on a regular basis until the next stage of the procedure. This first procedure lasted 5 h, and the blood loss was minimal.

The immediate recovery from this first procedure was uneventful, with a changing of the negative pressure dressing on the third postoperative day demonstrating a healthy wound with granulation tissue at its base, and an absence of purulent discharge. The patient remained in hospital, undergoing regular negative pressure dressing changes. On the 9th postoperative day, the patient spiked a temperature and became tachycardic; hence, there was a joint decision between the plastic and colorectal surgeons to perform an exploration under anesthesia. This showed known extensive hidradenitis on the groins bilaterally, and extensive induration of the upper thigh tissue, although there were no palpable collections. Multiple tracts were seen to be extending into the thigh proximally and medially, but none contained purulent material or obviously necrotic tissue. It was felt that there were no acute changes that provided an explanation for the spiked temperatures and the patient continued to recover. The patient was discharged home on the 31st postoperative day, remaining on antimicrobial therapy in the form of fluconazole and amoxicillin.

**FIGURE 3:**
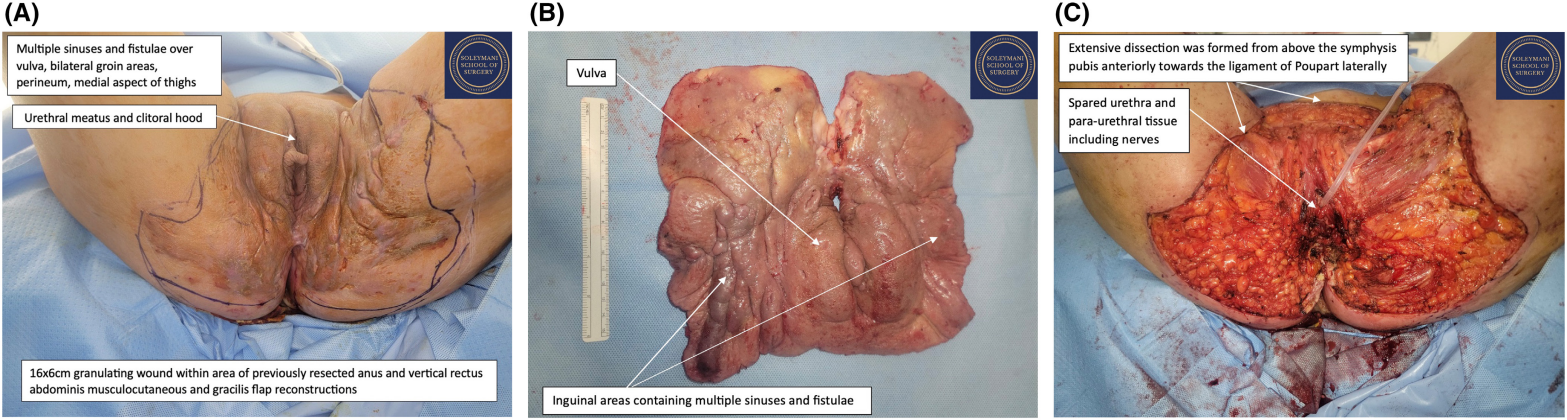
Second procedure: Radical bilateral anterior vulvectomy and posterior vaginectomy. (A) Examination under anesthesia, patient in modified Lloyd‐Davies position. (B) En‐bloc specimen comprising the vulva, lower vagina and bilateral groin structures including parts adductors longus, magnus and brevis and gluteus minimus, sparing the femoral vessels. (C) 25 cm × 28 cm defect with a central island of introitus with the external urethra and a vaginal stump.

The second procedure was performed 8 weeks after the first. Once anesthetized, the patient was examined, with multiple sinuses and fistulae found over the vulva and bilateral groin, and an absence of normal vulval tissue (Figure 3A). There was dense scar tissue over the site of the previous gracilis and vertical rectus abdominis musculocutaneous flaps. The cervix, urethra and paraurethra were all found to be normal. With the patient in a modified Lloyd‐Davis position, an extensive dissection was formed from above the symphysis pubis anteriorly towards the ligament of Poupart laterally. The femoral triangle was identified, with the dissection plane maintained above the cribriform fascia. The ischiocavernosus and bulbocavernosus muscles were then excised anteriorly, while posteriorly the previous vertical rectus abdominis musculocutaneous flap was excised along with the perineal body. The specimen comprising the vulva, part of the vagina and bilateral groin tissue was removed en‐bloc (Figure 3B), leaving a 25 cm × 28 cm defect with a central island of introitus with the external urethra and a vaginal stump (Figure 3C).

**FIGURE 4:**
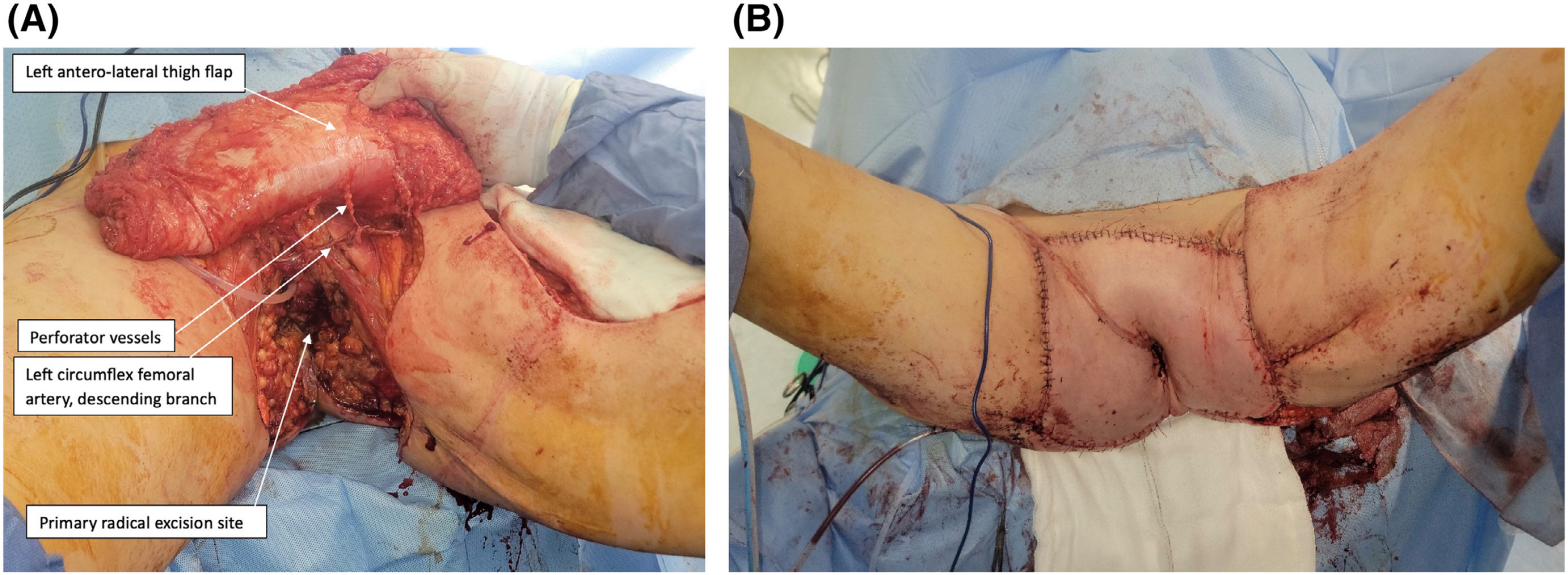
Second procedure: Flap reconstruction. (A) Reconstruction of the perineum using a left anterolateral thigh flap. (B) Central neo‐introitus with flap reconstruction.

The next stage of this procedure, undertaken by the plastic surgeons, was to reconstruct the perineum using a left anterolateral thigh flap (Figure 4A), based on four perforators from the descending branch from the lateral circumflex femoral artery. An incision was made along the anterolateral aspect of the thigh, parallel to the inguinal ligament, extending from the upper thigh to the knee. This incision divided the rectus femoris fascia, with the lateral flap raised to allow visualization of the muscular septum between rectus femoris and vastus lateralis, allowing for the intramuscular perforator dissection. The rectus femoris was separated using digital dissection and retracted medially, and four perforators were identified. These vessels were dissected until there was a pedicle length of 29 cm, to the origin of the profunda femoris artery and vein. A flap of 30 × 25 cm was raised and islanded on this pedicle with further extension of fascia lata. The flap was then tunneled under the rectus femoris muscle into the groin and the flap was inset into the defect in layers, with the fascia lata anchored to the bilateral groin, suprapubic, and perineal fascia. A central neo‐introitus was formed within the flap and parachuted down to the remaining stump of vaginal introitus, through which a urinary catheter was passed (Figure 4B).

To repair the donor area and part of the left proximal thigh, a split skin graft was harvested from the right thigh in three 4‐inch strips. This graft was then meshed at 1:1.5, expanding the area of the graft. A 6 cm × 7 cm area of graft was applied to the left proximal posterior thigh, with the remainder used to overgraft the left thigh flap donor site. Two Blake drains were inserted and left in‐situ in the donor site. The second procedure took 8 h and 45 min, with minimal estimated blood loss of around 200 mL. The patient was admitted to a high dependency unit postoperatively.

**FIGURE 5:**
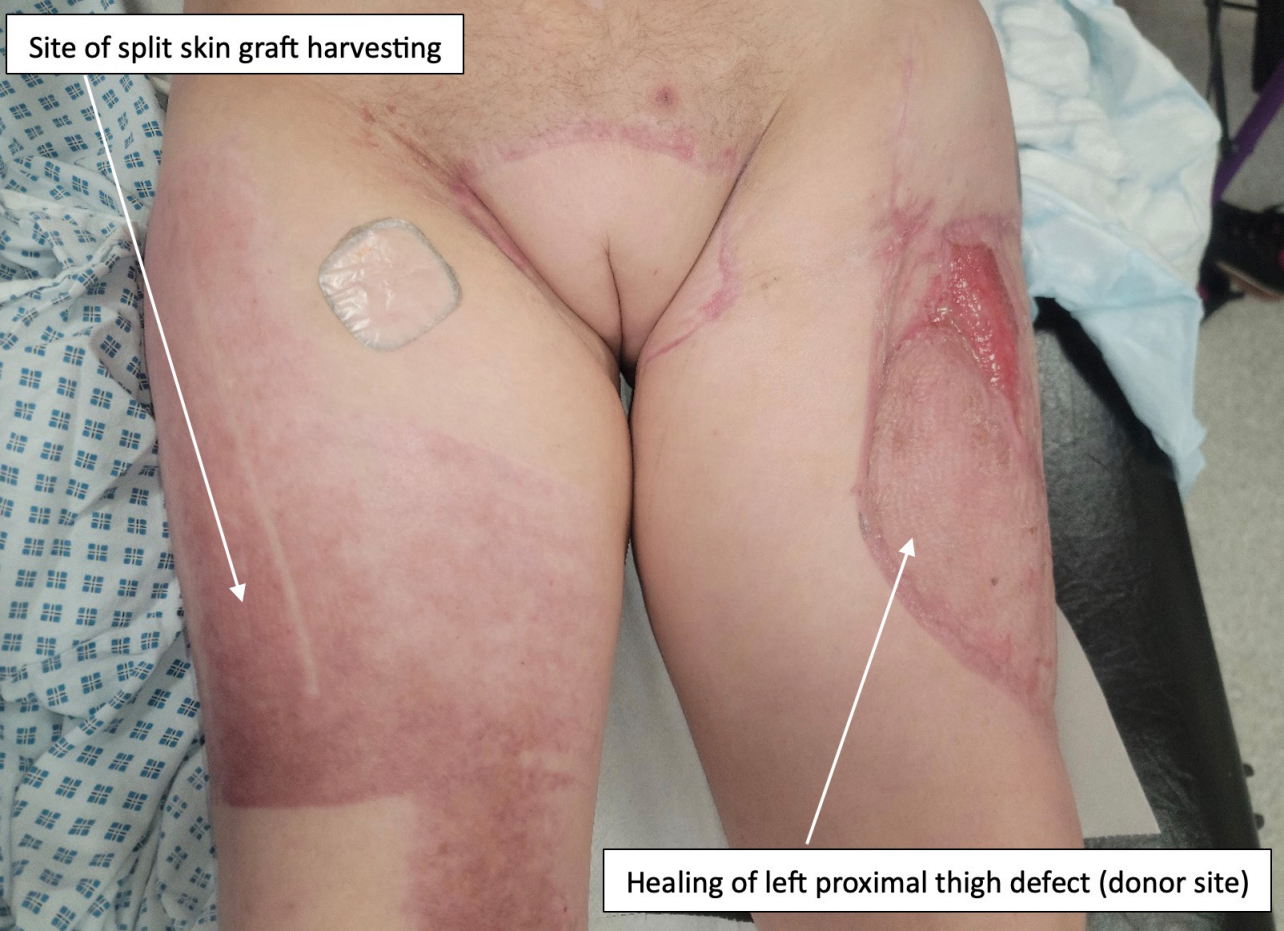
Six‐week postoperative review demonstrating good healing of the flap reconstruction, left proximal thigh donor site and skin graft harvesting site.

The patient's immediate recovery was straightforward initially. She underwent a dressing change under general anesthetic on the eighth postoperative day, which showed the flap to be healing well, good graft take to the left thigh, a small area of graft loss on the left buttock, a clean and granulating sacral wound, intact suture line, and the donor site to be clean with no signs of infection. On the ninth postoperative day, the patient developed abdominal pain, which was followed up by the general surgeons who examined her stoma, which was working normally. The IBD multi‐disciplinary team (MDT) discussed the patient and decided that in light of an MRI a few days prior showing thickening of the bowel wall, she should undergo an ileoscopy, which showed an ileal stricture and a mild–moderate flare of her Crohn's disease. Following a gastroenterology consult, she was started on 9 mg of budesonide daily for medical management of the Crohn's flare, with the potential for longer term treatment to be discussed at the IBD MDT. The budesonide was effective in treating the IBD flare, and the patient was able to begin mobilizing on the 14th postoperative day. She continued to recover well and was discharged home on the 32nd postoperative day. At 6 weeks postoperatively she was continuing to heal well (Figure 5), and the urinary catheter was removed 6 weeks postoperatively, with the patient regaining normal urinary function.

## 5 OUTCOME AND FOLLOW‐UP

The surgeons were able to successfully remove the hidradenitis suppurativa in the patient's perineum with wide margins, significantly easing her symptoms and preventing further septic episodes. She will continue to be followed up for her IBD by the IBD MTD, and input from other specialties such as dermatology will be required for consideration of medications such as biologic therapies to prevent a recurrence of the hidradenitis suppurativa in her perineum.

The patient was followed‐up by the plastic surgeons at monthly dressing clinics, which showed excellent wound healing in all areas. Apart from these reviews, the patient was doing her own dressing changes at home. There were two small areas of overgranulation seen 4 months postoperatively, which were treated with application of silver nitrate solution, and resolved. At a 7‐month review, the patient reported no longer having a need of dressings for her wounds, which were all nearly completely healed at this point. She will continue to be reviewed until the wounds are fully healed.

## 6 DISCUSSION

Hidradenitis suppurativa has been shown to be associated with other autoimmune diseases, notably IBD, and is characterized by the formation of sinuses, abscesses, and fistulae in intertriginous zones, causing severe pain and purulent discharge. This disease also has significant impacts on psychosocial aspects of health and quality of life, with patients at increased risk of depression, anxiety, and suicide.^12^

We have presented a particularly severe case of hidradenitis suppurativa that had failed to respond to previous medical treatments, necessitating excision of the coccyx, sacrum, vulva, posterior vagina, and lateral groin tissue, in a patient with a background of Crohn's disease, having undergone curative surgery in the form of a panprotocolectomy and permanent ileostomy. The surgical option for the hidradenitis suppurativa was very radical, hence ongoing discussion with the patient to discuss her wishes and priorities was essential, ensuring she was fully informed. The patient was made aware that there was an option to continue trying to manage this problem conservatively; however, she felt that her quality of life had deteriorated to a point that she was willing to undergo the significant surgery to remove the diseased areas of perineum and reconstruct them. The extent of the disease and involvement of a plethora of tissues meant that to fully resect the disease, and hence minimize the likelihood of its return, the involvement of multiple surgical specialties was required. The coccygectomy and partial sacrectomy were performed by the spinal team, the excision of the diseased regions of the perineum by the gynecological surgeons, and the reconstructive aspects were performed by plastic surgeons. Involvement of the general surgeons was also vital given the patient's medical and surgical history of Crohn's disease.

Maximal disease resection was required as this has been shown to minimize the risk of disease recurrence,^13^ with the MDT approach facilitating this. Ongoing input from other members of the MDT such as dermatologists will be important; the patient may require systemic treatment such as biologicals to continue to control her disease and prevent recurrence. There is a role for adalimumab in the treatment of hidradenitis suppurativa, however the patient is allergic to this, so biologicals targeting alternative molecules may be more appropriate. NICE guidance recommends a trial of secukinumab, which targets IL‐17A in patients unsuitable for treatment with adalimumab, and will publish their guidance on whether bimekizumab, targeting IL‐17A and IL‐17F could also be used in this setting. Another area of interest are monoclonal antibodies targeting IL‐1α, which have been suggested to be effective in patients ineligible for adalimumab.^14^, ^15^, ^16^

While an MDT approach can pose logistical challenges due to issues such as scheduling appointments for patients to be seen by multiple MDT members simultaneously, and scheduling of operations around multiple surgeons availability, in this case this approach was essential due to the complexity; a shared skillset was integral for successful surgery. A similar approach to surgical management of Crohn's disease has been described previously, with positive outcomes.^17^ In line with this, it has been shown that surgical MDTs can decrease peri‐^18^ and postoperative^19^ mortality. Due to the complexity of pelvic anatomy and the broad array of structures contained within it, MDT surgical collaboration has often been described for pelvic and other gynecological surgeries, resulting in successful patient outcomes.^20^, ^21^, ^22^ Overall, this approach was effectively utilized to maximize the chance of eradication of disease from the perineum, minimizing recurrence risk which should have a significant and positive impact on the patient's quality of life. Ongoing MDT input will be essential to continue to manage her hidradenitis suppurativa, with medical specialty involvement, such as dermatology, hopefully meaning that further surgical intervention is not necessitated.

## AUTHOR CONTRIBUTIONS

**Bea Harris Forder:** Investigation; methodology; project administration; visualization; writing – original draft; writing – review and editing. **Sabina Nistor:** Investigation; methodology; resources; supervision; writing – original draft; writing – review and editing. **Roman Mykula:** Conceptualization; data curation; investigation; methodology; project administration; resources; supervision; writing – review and editing. **Mark Bignell:** Conceptualization; data curation; investigation; project administration; resources; supervision; validation; writing – review and editing. **Hooman Soleymani majd:** Conceptualization; investigation; methodology; project administration; resources; supervision; validation; visualization; writing – review and editing.

## FUNDING INFORMATION

N/A.

## CONFLICT OF INTEREST STATEMENT

There are no conflicts of interest to declare.

## ETHICS STATEMENT

Not applicable.

## CONSENT

Written informed patient consent was gained for the submission of this case report.

## PERMISSION TO REPRODUCE MATERIAL FROM OTHER SOURCES

Not applicable.

## CLINICAL TRIAL REGISTRATION

Not applicable.

## Data Availability

Data sharing is not applicable to this article as no new data were created or analyzed in this study.
